# Systematic selection of competing metabolomics methods in a metabolite-sensory relationship study

**DOI:** 10.1007/s11306-021-01821-3

**Published:** 2021-08-25

**Authors:** Naser Davarzani, Carmen Diez-Simon, Justus L. Großmann, Doris M. Jacobs, Rudi van Doorn, Marco A. van den Berg, Age K. Smilde, Roland Mumm, Robert D. Hall, Johan A. Westerhuis

**Affiliations:** 1 Swammerdam Institute for Life Sciences, University of Amsterdam, Science Park 904, 1098 XH Amsterdam, The Netherlands; 2 Laboratory of Plant Physiology, Wageningen University and Research, Droevendaalsesteeg 1, Wageningen, 6708 PB The Netherlands; 3 Netherlands Metabolomics Centre, Einsteinweg 55, Leiden, 2333 CC The Netherlands; 4 Unilever Foods Innovation Centre, Bronland 14, Wageningen, 6708 WH The Netherlands; 5 DSM Food Specialties, Biotech Campus Delft, Alexander Fleminglaan 1, Delft, 2613 AX The Netherlands; 6 Wageningen Research (Bioscience), Wageningen University and Research, Droevendaalsesteeg 1, Wageningen, 6708 PB The Netherlands

**Keywords:** Metabolite-sensory relationship, Metabolomics, Sensory attributes

## Abstract

**Introduction:**

The relationship between the chemical composition of food products and their sensory profile is a complex association confronting many challenges. However, new untargeted methodologies are helping correlate metabolites with sensory characteristics in a simpler manner. Nevertheless, in the pilot phase of a project, where only a small set of products are used to explore the relationships, choices have to be made about the most appropriate untargeted metabolomics methodology.

**Objective:**

To provide a framework for selecting a metabolite-sensory methodology based on: the quality of measurements, the relevance of the detected metabolites in terms of distinguishing between products or in terms of whether they can be related to the sensory attributes of the products.

**Methods:**

In this paper we introduce a systematic approach to explore all these different aspects driving the choice for the most appropriate metabolomics method.

**Results:**

As an example we have used a tomato soup project where the choice between two sampling methods (SPME and SBSE) had to be made. The results are not always consistently pointing to the same method as being the best. SPME was able to detect metabolites with a better precision, SBSE seemed to be able to provide a better distinction between the soups.

**Conclusion:**

The three levels of comparison provide information on how the methods could perform in a follow up study and will help the researcher to make a final selection for the most appropriate method based on their strengths and weaknesses.

## Introduction

One of the new trends in food science is to relate the chemical composition of a food product to its sensory attributes at the level of individual metabolites or groups of metabolites (Seisonen et al., 2016). These relationships, that will improve our understanding of flavour formation due to processing and the supplementation of ingredients, are of great relevance to food design/formulation studies. Examples can be found for various products such as wine (Malherbe et al., 2013), olive oil (Procida et al., 2016), apples (Corollaro et al., 2014) and dairy products (Croissant et al., 2011).

The multivariate sensory profile of a food product can contain information on its appearance, flavour (aroma and taste), mouthfeel and texture which are usually assessed by an expert panel. In an experimental setup, expert panellists “measure” the products by scoring them for a number of pre-defined contrasting sensory attributes. The chemical content of the products can be measured by various analytical methods. Metabolomics is a common term used for the comprehensive characterisation of the molecules present in a biological sample (Cubero-Leon et al., 2014). Most importantly, metabolomic analyses can help to compare accurately the metabolite profile between groups of contrasting samples and thus to identify discriminatory compounds and subsequently enabling us to correlate chemotype determined by the metabolic space and phenotype as defined in the sensory space.

A large number of analytical techniques are suitable for measuring the broad range of metabolites. Mass spectrometry platforms are commonly used, coupled with gas chromatography (GCMS) or liquid chromatography (LCMS) as separation techniques. Within these two hyphenated approaches there is still a large variety of possibilities with respect to the choice for e.g. sample preparation and sample extraction (Thomsen et al., 2016). Furthermore, methodologies can be chosen which focus on a specific group of metabolites (targeted approach) or are aimed to detect as many metabolites as possible (untargeted approach) without any prior knowledge on the identity of the molecules (Alonso et al., 2015). The latter allows us to get a more comprehensive metabolite profile which can make associations with sensory data stronger.

The relationships between chemical compounds and the sensory impact may entail various challenges which play an important role in gaining a complete mechanistic understanding. These challenges are for example related to masking effects regarding individual compounds, nonlinearities or saturation effects and the presence of low abundant compounds which nevertheless have low odour thresholds. Such compounds play an overly-influential role in determining the aroma experience and are essential for the overall sensory characteristics (Diez-Simon et al., 2019; Yang et al., 2015). Moreover, current prediction models in the relationship between chemical composition and the sensory profile (Aprea et al., 2012; Calingacion et al., 2017; Esslinger et al., 2014) should be expanded to cover a larger number of compounds (untargeted approach) and multiple sensory attributes, as well as combining complementary analytical platforms. Knowledge of such approaches will help industry enormously in developing new product formulations with a predefined sensory profile.

Because of the large number of possible metabolomics methods to choose from in a chemical sensory relationship study, it is often difficult to decide which method to use to explore optimally the existing metabolite sensory relationships. In a pilot study, one could assess multiple methods to decide which one best suits the project at hand, particularly if the methods provide partly overlapping metabolite profiles. However, there are several issues that need to be taken into account while selecting the optimal metabolomics method in a given project.

In this paper, we introduce a methodology to select between potential metabolomics methods for a given metabolite-sensory relationship project using untargeted approaches. We use the term methods very generally—it could mean two different metabolomics platforms, but it could also mean two different separation columns in an LCMS, or two different sample extraction approaches. Such a decision between methods often has to be made in an early phase of the study based on only pilot data, yet the consequence of this decision will only become apparent in the larger follow up study, for which similar, but not the same samples are expected. The study discussed in this paper is the pilot study, but the decisions made from this study will be used to design the follow up study. In the larger follow-up study, new samples will be tested, which are, although related, different from the pilot study samples. Therefore, it is not sufficient to only focus on how well the sensory attributes could be predicted from the metabolite levels in the pilot study. Instead, the potential application of the two metabolomics methods for the new samples in the follow-up study, which are not yet available, has to be assessed. We will do this using various criteria such as the precision of the methods, number of features detected and the relevance of these features.

To introduce the methodology, we will use a tomato soup flavour study, where two different sample extraction techniques for volatile and semi-volatile aroma compounds were used in combination with GCMS: solid-phase micro extraction (SPME) and stir bar sorptive extraction (SBSE). These two approaches detect partly the same volatile compounds, but also are able to detect compounds that cannot be detected by the other method. Twenty-seven different tomato soup samples were prepared with different recipes according to a well-defined experimental design (Table 1). The soups were assessed for their aroma and taste profile by a trained sensory panel and analysed by SPME- and SBSE-GCMS. In the theory section we will use these two methods to explain the general approach.

**Table 1:** Compositional factor levels of the tomato soups used in this study

Soup nr	Tomato dosage	Oil type	Oil dosage	Yeast product	Yeast dosage	Included in QCs
1	Low	Olive	High	–	–	
2	Low	Olive	High	Maxarome	Low	
3	Low	Olive	High	Maxarome	High	
4	Low	Olive	High	Maxavor	Low	
5	Low	Olive	High	Maxavor	High	
6	Low	Olive	High	Maxagusto S-99	Low	
7	Low	Olive	High	Maxagusto S-99	Low	Included
8	Low	Olive	High	Maxagusto S-99	High	
9	Low	Olive	High	Maxagusto S-99	High	
10	Low	Olive	High	Maxagusto O-31	Low	
11	Low	Olive	High	Maxagusto O-31	Low	Included
12	Low	Olive	High	Maxagusto O-31	High	
13	Low	Olive	High	Maxagusto O-31	High	
14	Low	Olive	High	Maxagusto G-28	Low	
15	Low	Olive	High	Maxagusto G-28	High	
16	High	Olive	Low	–	–	
17	High	Olive	Low	Maxagusto S-99	High	Included
18	High	Olive	Low	Maxagusto O-31	High	Included
19	High	Olive	Low	Maxagusto G-28	High	Included
20	Low	Corn	High	–	–	
21	Low	Corn	High	Maxagusto S-99	High	
22	Low	Corn	high	Maxagusto O-31	High	
23	Low	Corn	High	Maxagusto G-28	High	
24	High	Corn	Low	–	–	
25	High	Corn	Low	Maxagusto S-99	High	Included
26	High	Corn	Low	Maxagusto O-31	High	Included
27	High	Corn	Low	Maxagusto G-28	High	Included

In the theory section we will lay down the methodology to select the most useful of the two analytical methods that best characterises the variation applied to the tomato soup flavour. The methodology comprises three parts that compare (1) the methods based on their analytical figures of merit, (2) the variation induced in the metabolite levels by the different products and, finally, (3) the quality of the relationship between the metabolite levels and the sensory attributes. In the results section we will present exemplary results of the comparison steps outlined above. Note that our aim is not to select the best method for this specific tomato soup project, but to outline a strategy that aids the decision making process.

## Theory

The comparison between the analytical platforms is carried out on three levels: comparison of analytical figures of merit; comparison of how well the measured metabolite levels are able to distinguish between the different products and finally, how well the variation in the metabolite levels over the products are able to model the variation in the sensory attributes. The information obtained from each of these levels can then be combined to make a final decision with respect to the most suitable method in the study.

### Comparison of analytical figures of merit (Level 1)

Traditionally, the quality of analytical platforms is assessed using analytical figures of merit such as reproducibility, linear range, sensitivity, selectivity etc. All these figures of merit are defined for single specific compounds. However, current analytical platforms provide levels of many metabolites simultaneously. The comparison of figures of merit between different methods can be performed in two ways: metabolite-independent and metabolite-dependent. The metabolite-independent approach calculates a specific figure of merit for all metabolites detected by the method. As an example, the reproducibility can be selected as the figure of merit of interest. Then, for each method, a density plot or a histogram gives information in general about the reproducibility of the whole set of metabolites in the two methods. Such a figure could provide information on the difference between platforms on how many metabolites can be measured with a specific reproducibility. In contrast, the metabolite-dependent approach considers only those metabolites that are measured in both methods (these are called common metabolites). For each of these metabolites, the figure of merit value is directly compared between the methods. If e.g. the reproducibility for most of these common metabolites is better in SPME, then that would be a good reason to select SPME over SBSE as the trapping method. Note that it is not necessary to know the identity of the metabolite, only that they are identical.

For the metabolite-dependent approach, the common metabolites must be defined first. A comparison of common metabolites detected in both methods will focus on their figures of merit: which method is able to detect these metabolites with the highest accuracy. The comparison of the metabolites that are unique will rather focus on which of the unique metabolites give a broader view of the differences between the products and whether they are able to improve the prediction of sensory properties of the products.

To find the common metabolites, we use the spectral information available for each metabolite, i.e., mass to charge ratios *m/z* (parent ions and ion fragments) and their corresponding relative intensities, together with its retention time index. In the following, we show for two mass spectrometry platforms how to find the common metabolites. The approach works as follows:

- For a given metabolite in SPME data, select those metabolites in the SBSE dataset for which their retention indices have a smaller difference than a user-defined threshold value. This is done to make a preselection of potential candidates that could be the same metabolite. In our example project, this value was set to two units, but can be adjusted depending on the methods used. If the methods are rather different, then this step could be omitted.
- Subsequently, the spectral similarities—expressed as cosine similarities (Stein & Scott, 1994)—between the given SPME metabolite and the candidate SBSE metabolites are calculated:
  1 $${S}_{AB}=\frac{{I}_{A}\cdot {I}_{B}}{\Vert {I}_{A}\Vert \Vert {I}_{B}\Vert }$$

Here, ${I}_{A}$ and ${I}_{B}$ represent vectors of the square root transformed intensities observed for the two metabolites that are being compared. Both, ${I}_{A}$ and, ${I}_{A}$ were zero padded for masses that were present in one of the mass spectra, but not in the other.

- The candidate SBSE metabolite with the highest similarity S_AB_ to the given SPME metabolite is selected. If S_AB_ is greater than a certain threshold, the two metabolites are considered to be identical. The value of this threshold can be set based on the known (annotated) metabolites from the two datasets.
- Repeat the analysis starting with each SBSE metabolite and use the same procedure to find the optimal SPME match. Only if this reverse analysis finds the same pair of metabolites, they are considered common metabolites.

### Comparison of ability to distinguish between product preparation differences (Level 2)

In projects that aim to build relationships between sensory and metabolic profiles of a product, usually a set of contrasting “pilot products” is developed based on an experimental design strategy in which certain ingredients are added or certain process steps have been taken. The formulations of the pilot products should be defined with the aim of ensuring sufficient variation in their metabolic as well as in their sensory profile. In this second level we will analyse the relationships between the metabolite levels of the products and the compositional factors, i.e. the different ingredients and processing steps that are expected to influence the molecular composition of the products.

If the experimental design from which the samples are made contains qualitative factors (e.g. tomato high or low, yeast product A, B or C, corn oil or olive oil), this is a discrimination problem between the products that are made in a specific manner and the other products. Such a discrimination problem can be explored in a univariate manner, where for each metabolite we test its performance in discriminating between the groups, as well as in a multivariate manner where also the correlation between the metabolite levels are considered in the discrimination model. Multivariate discrimination methods that can be used to quantify how well the specific platform is able to discriminate between the two groups of products are for example PLSDA, SVM and random forest classifiers (Lee et al., 2018; Liu et al., 2013). If the experimental design used quantitative factors (e.g. different concentrations of yeast product), then this could be dealt with using a regression approach. In such a case the prediction error for the different concentration levels should be small enough to distinguish between the samples. Prediction errors can be addressed using root mean squared error of predictions (RMSEP) or Q^2^.

### Comparison on ability to predict sensory attributes (Level 3)

In the final level of comparison we explore how well the metabolite levels of the different platforms are able to predict the sensory attributes of the different products. Here, a predictive multivariate model is built between metabolite levels and sensory profiles to see which platform is best able to predict the sensory profile as well as which of the metabolites has important roles in these models. For this step, a multivariate regression model has to be used as the sensory attribute is usually a quantitative feature. Examples for such models are SVR (Sugimoto et al., 2010), PLS (Grabež et al., 2019), penalised multivariate regression such as the LASSO or Elastic Net Regression, or Random Forest (Welzenbach et al., 2016).

Note that one could assume that only level 3 is important as this aspect reflects precisely the goal of the project, namely the metabolite-sensory relationship. However, the comparison is done on a pilot scale, and should provide more information to select new samples for the larger study. Therefore, the check whether there is sufficient variability between the samples that can be observed with the platform (level 2) is important as well as whether the platform is able to quantify the measured metabolites with sufficient quality (level 1).

## Experimental

The specific case study we will use in the paper to demonstrate the methodology is from a project in which the volatile metabolites in 27 different tomato soup products had to be related to various sensory attributes. The tomato powders (Unilever R&D, Wageningen, The Netherlands) consisted of: tomato powder, sucrose, roux, starch, oil, salt, lemon juice, pepper and yeast-derived flavour products (DSM, Delft, The Netherlands) (or nothing, in case of blanks) (Table 1). The main difference between the powders were the type of oil used (corn vs. olive), the tomato dosage (high vs. low), the type of yeast product and its dosage (high vs. low). The soups were prepared by stirring 70–99 g of the tomato mix powder into 1 L of boiling water. The exact amounts depended on the formulation. The soups were gently simmered for 5 min and occasionally stirred.

### Sensory analysis description

Investigating the effects of odour, flavour, mouthfeel, aftertaste, and afterfeel requires an extended evaluation of the products and this was done by Quantitative Descriptive Analysis (QDA). During the QDA measurement, the intensities of the attributes were obtained by EyeQuestion (Logic8), using unstructured line scales ranging from 0 to 100. A very experienced (> 10 years) group of panellists (*n* = 14) assessed the 27 different products divided over 4 sessions. For each session, all 27 products were freshly prepared, and offered one-by-one to the panellist according to an incomplete, balanced design that was specifically developed to assess all products in each session. The products were stored in a holding cabinet at 60 °C prior to sensory analysis. Fifty mL of each tomato soup was provided to the panellists in a white polystyrene cup. Overlap between the sessions was ensured by having replicate samples across the different sessions which were tasted by several panellists.

The variation in assessment of the different products varied greatly between the different assessors. To correct for difference between assessors in terms of level effect and scaling effect, a standardisation of the sensory data was used. The intensity assessed for attribute *k* by panellist *i* for product *j* (${y}_{ijk}$) is standardised to ${\tilde{y }}_{ijk}$ as follows:

2 $${\tilde{y }}_{ijk}=\frac{{y}_{ijk}-{\overline{y} }_{ik}}{{s}_{ik}}$$

where ${\overline{y} }_{ik}$ is the mean intensity for attribute *k* and panellist *i* of all soups and ${s}_{ik}$ is the standard deviation for attribute *k* and panellist *i* of all soups (Romano et al., 2007). This standardisation corrects for panellists that, on average, give higher or lower intensity values for all products or use different ranges of the rating scale than the average panellist (e.g. from 40 to 55 instead of from 20 to 70). After standardisation, the attribute intensities were checked for normality. In cases of skewed distributions, a transformation can be applied, but for the attributes discussed in this paper, this was not necessary.

### Metabolomics platforms

For the determination of the metabolite levels, two different approaches (SPME and SBSE) were used in which the extraction of the metabolites from the samples differed. The two approaches were discussed in detail in an earlier paper (Diez-Simon et al., 2020). After extraction, analytes were thermally desorbed and analysed by GCMS. The same GCMS instrument and settings were employed for both extraction techniques. Extraction and analysis of both techniques followed the same procedure as described before by Diez-Simon et al. (2020). The only variation from the previous study was the temperature used, in both SPME and SBSE, to desorb the analytes into the GC. In this study 280 °C, instead of 250 °C, was applied in order to extract the less volatile, high molecular weight compounds which are present in the complex tomato soup matrices.

### Experimental setup

For both techniques, a set of 27 tomato soups were analysed in a randomised way. An empty glass vial and a blank (water) sample were measured at the beginning of each sequence. Quality control samples (QCs), which were a mix of a few selected tomato soup samples (see Table 1), were repeatedly analysed along the sequence to test the performance of the analysis. Five QCs were analysed in the SPME series, once every six samples and four QCs for SBSE, after every ninth sample. The raw GCMS data were processed using an untargeted metabolomics approach, which has also been described in detail before (Diez-Simon et al., 2020).

### Data analysis methods

For the level 2 classification models, PLSDA was used to discriminate between samples containing the yeast product O31 (7) and all other samples (20), as well as a to discriminate between samples made with olive oil (19) vs samples made with corn oil (8). The PLSDA models were generated using the *mixOmics* package (R 4.0) on the square root transformed and zero-centered mass spectrometry data; model performance was evaluated using a fivefold cross validation procedure. The discrimination power was quantified using the balanced error rate (BER), which is defined as:

3 $$BER=1-\frac{1}{2}\left(\frac{tp}{tp+fn}+\frac{tn}{tn+fp}\right)$$

Here, tp, tn, fp and fn represent the number of true positives, true negatives, false positives and false negatives. Selectivity ratios were calculated for each feature according to Rajalahti et al. (2009).

For level 3 predictions of intensity.od and umami.fl as example sensory attributes, an elastic net calibration model was used (*glmnet* package, R 4.0). The elastic net mixing parameter $\alpha$ and the regularisation parameter $\lambda$ were optimised simultaneously by minimising the prediction error in a leave-one-out cross-validation procedure. The prediction error was quantified as the mean squared error (MSE), which is defined as:

4 $$MSE=\frac{\sum {\left({y}_{i}-{\widehat{y}}_{i}\right)}^{2}}{n(y)}$$

Here, ${y}_{i}$ represents the sensory attribute value for sample i, ${\widehat{y}}_{i}$ represents its prediction and $n(y)$ represents the number of samples.

## Results

### General analysis results

For the two trapping techniques the number of detected metabolites after processing of the data was different. With SPME a total 331 metabolites were detected, while 482 metabolites were detected with SBSE. For each of the detected metabolites a mass spectrum is available. By matching the obtained mass spectra and retention indices to either our authentic reference standards or those in the NIST17 Mass Spectral library (v.2.3), 100 and 110 metabolites were annotated in the SPME and SBSE datasets, respectively. Before analysis on how well the metabolomics data is able to distinguish between products and to relate to the sensory attributes, a square root transformation was applied to correct for heteroscedasticity.

In this paper we will focus on two sensory attributes, the odour intensity (intensity.od) and umami flavour (umami.fl). The former is an attribute highly affected by the volatile metabolites, while the latter has been described to be mainly related to non-volatile glutamic acid and some nucleotides, compounds that were not targeted with the current analysis protocols. Statistical testing of only the sensory data yielded F-test values for differences between products of 3.5E^−13^ for odour intensity and 0.03 for umami flavour. Thus, there is sufficient variation in odour intensity, but limited variation between umami levels of the 27 soup products.

### Results of methodology

#### Level 1

For the first level of the comparison, we selected to analyse the reproducibility using the relative standard deviation of the processed metabolite data. The reproducibility was obtained from the QC samples that were measured throughout the batches. Furthermore, we used the concentration range (defined as the difference between maximum and minimum intensity value) found for the common metabolites in the soup product samples. A larger range across these soup product samples relates to a higher sensitivity for those compounds as the product samples in both methods are the same.

For the metabolite independent approach we calculated the relative standard deviation (RSD) which is the ratio of the standard deviation over the mean value obtained from multiple measurements of the QC sample in both methods. For the SPME method, five QC measurements were available while for the SBSE method four QC samples were available.

**Fig. 1:**
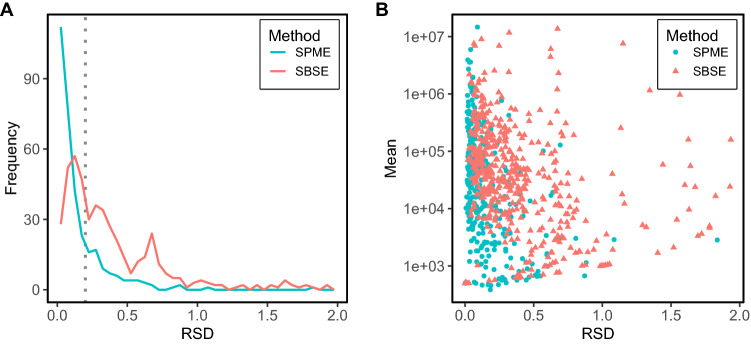
Metabolite-independent analyses on QC measurements. **A** Histogram of relative standard deviations for metabolites measured using SPME and SBSE. **B** Mean intensity over QC samples of the metabolites in logarithmic scale as a function of their RSD

Histograms of the RSD in SBSE and SPME are shown in Fig. 1A. The dotted line in the density plot indicates an RSD value of 0.20, which is sometimes used as a threshold for whether metabolites will be reported or not (Siskos et al., 2017). The fraction of metabolites that were measured with a low relative standard deviation (RSD) is much higher for SPME than for SBSE. A large number of metabolites detected using SPME has an RSD value below 0.20, pointing to many metabolites that can be quantified rather precisely, whereas for SBSE, a much larger group of metabolites were measured with an RSD > 0.2. Moreover, many of the SPME metabolites with small RSD have relatively high mean values (Fig. 1B), and thus could be very useful for the distinction between the soups.

##### Metabolite-dependent comparison

In the second part of level 1 comparison we focused on the common metabolites found using both methods. To find metabolites that are present using both approaches, both the mass spectral information as well as their retention information were used, as was discussed in Sect. 2.1.

The procedure selecting common metabolites in the two extraction methods was validated using the annotated metabolites in the SPME and SBSE datasets. Based on the annotated features in both datasets, the cosine similarity threshold was set to 0.65. Out of the 61 annotated compounds that were present in both datasets, 59 were correctly detected to be common whereas zero out of 88 non-common compounds were falsely detected to be common. The two compounds that were not found to be common by our approach were rejected because of a too large difference in retention index due to transfer issues for these two compounds which eluted at the beginning of the chromatogram.

**Fig. 2:**
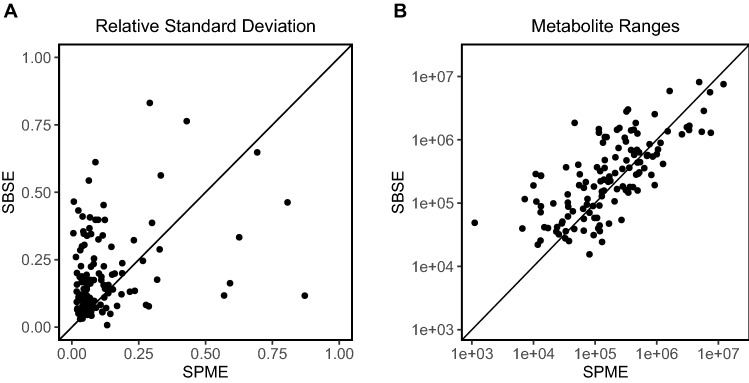
Metabolite-dependent analyses on common metabolites. **A** Relative standard deviations of common metabolites between QC measurements, compared between SPME and SBSE. **B** Ranges of common metabolites compared between SPME and SBSE represented in log scale

In addition to the 61 annotated common metabolites, application of the matching procedure followed by manual verification yielded another 69 metabolites that are common but not annotated. Using these 130 common metabolites, the RSD and the range comparison for the SPME and SBSE methods were performed. The RSD scatterplot (Fig. 2A) shows more dots above the diagonal line, indicating that many of the common metabolites have higher RSD values for SBSE than for SPME. The range of the common metabolites is quite similar for both SPME and SBSE methods. Thus, although the reproducibility seems better for the SPME method, the sensitivity for the metabolites that are in common is similar for both methods.

#### Level 2

For the second level of comparison we used discrimination methods to investigate how well the metabolites were able to distinguish between groups of products that were produced using different recipes. This can be applied using univariate approaches that present how many of the metabolites are able to distinguish between the groups by themselves, or in a multivariate manner where combination of metabolite levels can be used to discriminate between the groups of products. For the univariate approach we calculated the Point-biserial correlation (Sheskin, 2011) between the group indicator variable and the metabolite levels. The Point-biserial correlation is comparable to the Pearson correlation coefficient, but corrects for the fact that the group indicator variables have a dichotomous structure (Sheskin, 2011). For multivariate discrimination, we applied Partial Least Squares Discriminant Analysis (PLSDA). This method uses a latent variable model (using many correlated metabolites simultaneously) to discriminate the two groups in the data.

To show how well the two different platforms detect metabolites that can discriminate soups made with different recipes we considered two exemplary compositional factors. In the first example we compared soups containing the process flavour O31 vs all other soup types, and in the second example, soups produced using olive oil were compared to soups made using corn oil. Note that the metabolomics data was square root transformed.

**Fig. 3:**
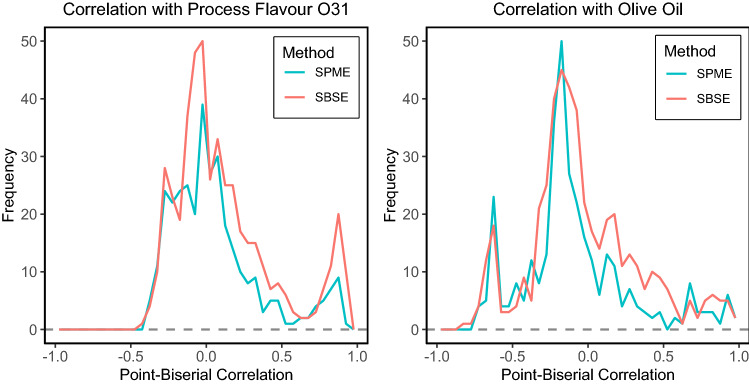
Univariate analysis of the relationships between metabolite levels and compositional factor levels. Histograms of point-biserial correlation between the metabolite levels of the SBSE and SPME platforms and the group indicator of O31 vs the other soups (left) and between soups made with olive oil vs corn oil (right). Here positive correlation means the peak is higher for olive oil samples, while negative correlation means the peak is higher for corn oil samples

In the point-biserial correlation coefficient histogram plots for the O31 vs others discrimination (Fig. 3, left) and the olive oil vs corn oil discrimination (Fig. 3, right), the highest peaks were found near a correlation of zero, indicating that most metabolites in both platforms are not correlated to the O31 status nor to the oil status. However, in the O31 correlation histogram plot we see a small peak around a correlation of 0.9 for both platforms, indicating some metabolites with a high positive correlation with O31. For SBSE this peak is much higher than for SPME, suggesting that the SBSE method data contains more metabolites indicative for O31 than the SPME method. For the oil status, the number of metabolites with a high absolute correlation value is much lower, and virtually no difference can be observed between SPME and SBSE. The histogram also reveals a distinct group of compounds with a correlation of around − 0.65 that were only present in samples with high tomato concentration in combination with corn oil. A possible explanation may be that these volatiles are only present in corn oil and more released in samples with low oil concentrations. Previous studies have shown the release of fat-soluble flavour compounds is dependent on the oil content (Patana-anake et al., 2015). Further studies are needed to explore such interaction effects.

For the multivariate PLSDA models, the O31 status and also the olive oil status were predicted from the metabolite levels of SBSE or SPME platforms. To quantify the model performance, we use the Balanced Error Rate (BER), which is the average of the False Positive Rate and the False Negative Rate (Rohart et al., 2017), and should be as small as possible. The BER is useful when the groups in the discrimination model are of unequal size, which is the case for our examples. The BER is not a relative measure, thus it can be used to compare the SBSE and SPME models for the same response (e.g. O31) but not for comparing models for different responses. To indicate metabolite importance in the PLSDA model we used the selectivity ratio (Rajalahti et al., 2009).

**Fig. 4:**
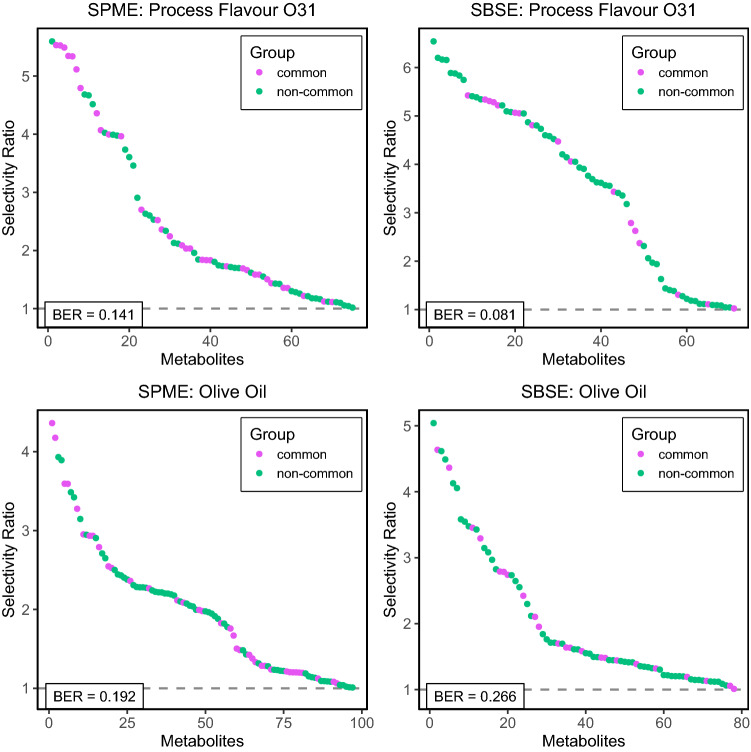
Multivariate analysis of the relationships between metabolite levels and compositional factor levels using PLSDA. The plots show selectivity ratios and balanced error rates (BER) of the PLSDA models for process flavour O31 (top) and olive oil status (bottom) using the SPME (left) and the SBSE (right) metabolite data.The selectivity ratio (only variables with a selectivity ratio above 1 are shown) reflects the discriminatory power of each variable, whereas the BER is a measure for the overall discriminatory performance of the models

Selectivity ratios for metabolites in the SPME and SBSE data, as calculated from the PLSDA models, and the balanced error rate (BER) values are provided in Fig. 4. The pink coloured dots represent metabolites which are in common in both methods, while the green ones are unique per method. It can be observed that the O31 status is predicted better using SBSE than using SPME, as indicated by the lower BER value. For the olive oil prediction, the SPME model performs slightly better. Inspection of the metabolite selectivity ratios reveals that for the SBSE models many of the highly predictive metabolites are unique to the SBSE dataset. Thus, the use of SBSE enables quantification of a number of metabolites that are discriminative for the O31-containing soups as well as the olive oil containing soups, but do not appear in the SPME dataset.

The advantage of the multivariate approach (PLSDA) over the univariate approach is that relationships between the independent variables (metabolite levels) can be taken into account and thus noise can be handled better. In the application here, both approaches deliver very similar results with regard to the features that are deemed relevant for the classification, but PLSDA additionally provides a clear measure of classification performance.

#### Level 3

The third level of comparison between the two platforms is to assess which set of metabolites is better able to predict the sensory properties of the different soups. A multitude of sensory attributes were assessed by the expert panel. We selected two different attributes (odour intensity and umami flavour) to see how well these can be predicted using metabolite level data from the SPME or SBSE platforms. Odour intensity is expected to relate strongly with volatile metabolites while umami flavour is not.

As an example, we used an elastic net regression model approach to make predictive models for the selected attributes using the square root transformed metabolite data from SPME and SBSE. The elastic net model is a penalised regression model that selects only metabolites that have a sufficiently large effect on the prediction of the attribute. The tuning parameters for the elastic net models were optimised for each platform using cross validation. The mean squared error (MSE) of the prediction as a measure of model performance was obtained using cross validation. The MSE is not a relative measure as it depends on the size of the response value. Therefore, MSE values of models for the same response can be compared, but MSE values for different responses cannot.

**Fig. 5:**
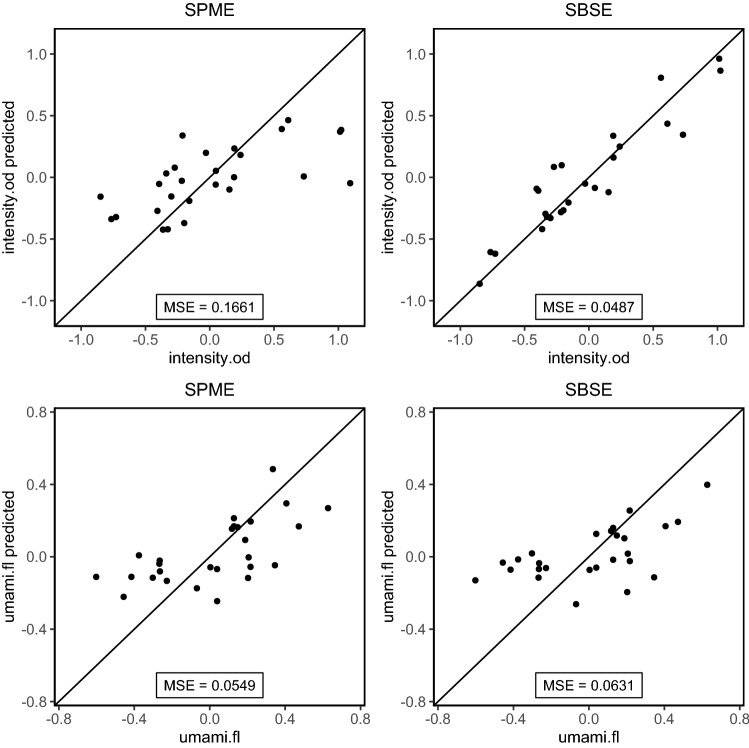
Performance of Elastic Net models in predicting odour intensity (top) and umami flavour (bottom) from SBSE (left) and SPME (right) data. The diagonal lines represent the predicted = observed line. The mean squared error (MSE) is a measure for the quality of prediction

In this step, the exact nature of the sensory panel data comes into play. While for many multivariate regression methods a variance-stabilising transform of the independent variables is advisable, certain restrictions may also apply to the response variable on this level (sensory panel data). Transformations can aid in reducing skewness and removing heteroskedasticity from the response variables. In this study, no such adjustments were required for the selected sensory attributes (Fig. 5).

The observed values for odour intensity could be well predicted using SBSE, while the prediction performance using SPME is limited. Metabolites most important to predict odour intensity using SBSE were 2,6-diethyl-Pyrazine (Koehler et al., 2006), Methylbutanal and Eugenol (Patanake-anake et al., 2015). For umami flavour, as expected, both models had difficulty making good predictions.

## Discussion and conclusion

In this paper we have provided a methodology to decide between two analytical methods as to which one would be most useful in a metabolite-sensory relationship study. Such a decision often has to be made in an early phase of a study where only pilot data are available while decisions have to be made for a larger study. In the larger study, new products might have to be tested which are different from the products included in the pilot study, and therefore it is not sufficient to only focus on how well the sensory attribute could be predicted from the metabolite levels using only the pilot products. The potential application of the two metabolomics methods for new products has to be assessed.

We have tackled this challenge by analysing the pilot results on three levels.

o Level 1: How well are the two methods able to measure the metabolites they can detect? For this we looked at the precision of the methods using QC samples. We have shown this can be done in an overall manner using a metabolite independent approach, but also in a direct comparison using only the metabolites that were measured in both metabolomics methods (common metabolites).
o Level 2: How well are the two methods able to detect differences between the products? Here we assumed that the products in the pilot study were made using an experimental strategy, such that the experimental factors could be tested for differences between their levels, e.g. compare all products made using olive oil vs all products made using corn oil. It is expected that in the follow-up study, similar experimental strategies will be used, but that the specific products made using those strategies will be different. Therefore, it makes more sense to focus on how well the different strategies can be distinguished using the metabolomics methods.
o Level 3: How well are the two methods able to provide metabolite levels that are able to predict sensory attributes of the pilot products? Of course, for the larger study this is the relevant question, and we would like to choose the method best able to make this prediction. However, we have to make a decision based on a small set of pilot samples.

In the example study of the 27 tomato soups reported in this paper, the results are not always consistently pointing to the same method as being the best. SPME was able to detect metabolites with a better precision, SBSE seemed to be able to provide a better distinction between the 27 soups and was also better in predicting some of the attributes. This could be due to the larger number of metabolites measured with SBSE that were not detected using SPME, and which also seemed to be relevant for the metabolite sensory relationship in the tomato soup project.

Depending on the final goal of the larger follow-up study, a different conclusion could be obtained from the results provided. If the study aims at precise quantification e.g. when a comparison with a product of a competitor is necessary, the many metabolites with low RSD could be useful and favour SPME. When new soup products have to be developed with specific sensory properties, the SBSE method could perform better.

Concluding, there are many aspects to consider when designing a study relating sensory characteristics to metabolomics data. We have provided a framework for designing such studies that addresses three major aspects of such a study: (a) the reproducibility of the possible platforms to use, (b) the diversity of the platforms with regard to the products to be tested and, finally, (c) initial analysis to confirm whether the platform has predictive relevance for the sensory characteristics. We recommend using this framework for future sensory studies since it provides a systematic strategy for analysing screening experiments which may be used to design a full sensory study.

## Data Availability

The datasets generated during and/or analysed during the current study are not publicly available because they are still under exploration but are available from the corresponding author on reasonable request.
